# In Search of Differential Inhibitors of Aldose Reductase

**DOI:** 10.3390/biom12040485

**Published:** 2022-03-22

**Authors:** Francesco Balestri, Roberta Moschini, Umberto Mura, Mario Cappiello, Antonella Del Corso

**Affiliations:** 1 Biochemistry Unit, Department of Biology, University of Pisa, Via S. Zeno 51, 56127 Pisa, Italy; francesco.balestri@unipi.it (F.B.); roberta.moschini@unipi.it (R.M.); umberto.mura@unipi.it (U.M.); antonella.delcorso@unipi.it (A.D.C.); 2 Interdepartmental Research Center Nutrafood “Nutraceuticals and Food for Health”, University of Pisa, 56127 Pisa, Italy

**Keywords:** AKR1B1, aldose reductase, aldose reductase inhibitors, aldose reductase differential inhibitors, diabetes, oxidative stress, inflammation

## Abstract

Aldose reductase, classified within the aldo-keto reductase family as AKR1B1, is an NADPH dependent enzyme that catalyzes the reduction of hydrophilic as well as hydrophobic aldehydes. AKR1B1 is the first enzyme of the so-called polyol pathway that allows the conversion of glucose into sorbitol, which in turn is oxidized to fructose by sorbitol dehydrogenase. The activation of the polyol pathway in hyperglycemic conditions is generally accepted as the event that is responsible for a series of long-term complications of diabetes such as retinopathy, cataract, nephropathy and neuropathy. The role of AKR1B1 in the onset of diabetic complications has made this enzyme the target for the development of molecules capable of inhibiting its activity. Virtually all synthesized compounds have so far failed as drugs for the treatment of diabetic complications. This failure may be partly due to the ability of AKR1B1 to reduce alkenals and alkanals, produced in oxidative stress conditions, thus acting as a detoxifying agent. In recent years we have proposed an alternative approach to the inhibition of AKR1B1, suggesting the possibility of a differential inhibition of the enzyme through molecules able to preferentially inhibit the reduction of either hydrophilic or hydrophobic substrates. The rationale and examples of this new generation of aldose reductase differential inhibitors (ARDIs) are presented.

## 1. Introduction

Aldose reductase (E.C. 1.1.1.21) is a cytosolic enzyme which belongs to the aldo-keto reductase superfamily that includes about 190 enzymes capable of reducing different carbonyl substrates including sugars and products deriving from lipid peroxidation [1]. Aldose reductase has been classified within the aldo-keto reductase family as AKR1B1. This enzyme has been found in most mammalian cells, even though its distribution is not uniform among the various tissues: high levels of AKR1B1 are present in lens, sciatic nerve, testicle, heart and cornea; while organs such as liver, stomach, spleen, lung, small intestine and colon express low levels [2]. AKR1B1 catalyzes the NADPH dependent reduction of a series of aldehydic compounds to the corresponding alcohols. The catalytic mechanism of AKR1B1 is a sequential ordered one, in which NADPH binds to the enzyme before the aldehydic substrate; subsequently, after the reduction of aldehyde into alcohol, the latter is released before the cofactor [3]. The enzyme has an α/β barrel structure [4], with two auxiliary helices: the H1 α-helix responsible for the binding of the cofactor and the H2 α-helix located in the C-terminal portion [5]. NADPH is sequestered from the solvent by a series of residues ranging from Gly-213 to Ser-226. This region changes its own conformation depending on whether or not the NADPH cofactor is present and it can assume an “open” conformation, in the absence of NADPH or a “closed” conformation in the presence of the cofactor [5]. In the active site of the enzyme two regions can be identified: a rigid region, the so-called called “anion-binding pocket” containing residues Trp-20, Val-47, Tyr-48, His- 110, Trp-111 and a more flexible hydrophobic region, called “specificity pocket”, containing residues Thr-113, Phe-115, Phe-122, Cys-303 and Tyr-309 [6,7,8,9,10,11].

## 2. Aldose Reductase and the Polyol Pathway

**Figure 1:**
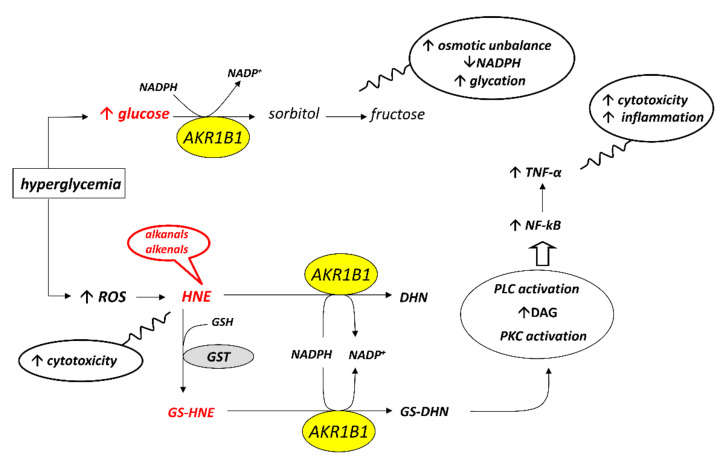
The multifaceted activity of AKR1B1. HNE: 4-hydroxy-2,3-nonenal; DHN: 1,4-dihydroxy-2,3-nonene; GS-HNE: 3-glutathionyl-4-hydroxy-nonanal; GS-DHN: 3-glutathionyl-1,4-dihydroxinonane; DAG: diacylglycerol; GST: glutathione S-transferase; NF-kB: nuclear factor kB; PKC: protein kinase C; PLC: phospholipase C; TNF-α: tumor necrosis factor α.

AKR1B1 is the first enzyme of the so-called polyol pathway, which converts glucose to sorbitol; the latter is then oxidized, to fructose by the enzyme sorbitol dehydrogenase through an NAD^+^ dependent reaction (Figure 1).

Due to AKR1B1’s low affinity for glucose, under normoglycemic conditions, glucose preferentially enters the glycolytic pathway and the hexose monophosphate shunt so that less than 3% of glucose is converted to sorbitol by AKR1B1 [12]. However, under hyperglycemic conditions, such as those in diabetic subjects, the hexokinase is saturated with a consequent increase of the intracellular levels of glucose; thus, approximately 30% of glucose is channeled into the polyol pathway to be converted into sorbitol [12]. The latter cannot diffuse out passively and accumulates generating an osmotic imbalance that induces the diffusion of water into the cell. The resultant swelling and electrolyte imbalance lead to cell damage. In addition to the accumulation of sorbitol, the increased flow of glucose through the polyol pathway causes a decrease in the levels of the cofactors NADPH and NAD^+^, thus determining an unbalance of the correct NAD^+^/NADPH ratio. Since NADPH plays a key role in protecting against the toxicity of reactive species of oxygen (ROS), acting as a cofactor of antioxidant enzymes such as glutathione reductase, the consumption of NADPH causes an increase of cell oxidative stress [13].

Furthermore, NADPH is also a cofactor of nitric oxide synthase; thus, under hyperglycemic conditions, the competition between AKR1B1 and nitric oxide synthase for the cofactor NADPH may limit nitric oxide production causing vasoconstriction, ischemia and slowing of nerve conduction [14]. At the same time, the increase of NADH due to the high activity of the polyol pathway could lead to increased synthesis of diacylglycerol. The latter is an activator of protein kinase C (PKC) that in turn induces the activation of Mitogen-Activated Protein Kinases (MAPKs). MAPKs phosphorylate transcription factors thus generating an alteration of normal gene expression, with the possible onset of apoptotic phenomena and development of vascular complications [15,16].

Another deleterious effect linked to the increase in the activity of the polyol pathway concerns the accumulation of fructose, which is considered a glycating agent responsible for the formation of Advanced Glycated End products (AGEs) [17]. The presence of AGEs contributes to the development of diabetic complications such as the formation of cataracts and nephropathy. The glycation products are generated through a non-enzymatic reaction (Maillard reaction) between the carbonyl group of glucose or fructose and amino groups of proteins. This reaction forms Schiff bases, which can rearrange to the so-called Amadori products. These early products eventually undergo irreversible modifications generating AGEs [18]. AGEs can bind various receptors on the cell surface and stimulate the production of reactive species of oxygen, the increase of cell permeability and inflammation [17,19].

## 3. Aldose Reductase, a Detoxifying Enzyme

Besides its role in the polyol pathway, AKR1B1 is involved in detoxification processes under oxidative stress conditions. Lipid peroxidation is a typical consequence of oxidative stress and generates hydrophobic aldehyde compounds such as alkanals, alkenals and hydroxyalkenals capable of inducing cell damage through modification of proteins and nucleic acids [20]. One of the most relevant products of lipid peroxidation is 4-hydroxy-2,3-nonenal (HNE) [20], which is considered a classical marker of oxidative stress. HNE is a very reactive molecule due to the presence of a carbonyl group on C-1, a double bond between C-2 and C-3 and a hydroxyl group on C-4. This structure makes HNE able to carry out a nucleophilic attack on various targets, such as lysine, cysteine and histidine of proteins or nucleic acids [20]. The formation of HNE-protein adducts has been shown to have a cytotoxic effect [21]. The effects of HNE on cells depend on its intracellular concentration: while low HNE concentrations promote proliferation in some cell types [22], high concentrations of this compound block cell cycles. HNE can bind to the Fas receptor thus activating the Apoptosis Signal-regulating Kinase 1 (ASK1) and the c-Jun N-terminal Kinases (JNKs) which induce phosphorylation and activation of caspase 3. On the other hand, the intracellular HNE regulates the expression of apoptotic genes through interaction with p53 [23]. Indeed, some studies have highlighted the role of HNE as a mediator of apoptosis induced under oxidative stress conditions, such as those determined by H_2_O_2_ and UV rays [22,23], and high concentrations of HNE have been found in several cardiovascular and neurodegenerative diseases [24]. Due to its deleterious effects, the intracellular concentration of HNE needs to be controlled. HNE can be detoxified through non-enzymatic and enzymatic pathways [25]. AKR1B1 can participate in the detoxification of HNE since it catalyzes the reduction of the carbonyl group of HNE, with the formation of 1,4-dihydroxy-2,3-nonene (DHN) (Figure 1). Indeed, some in vitro studies have shown a greater affinity of the enzyme for lipid peroxidation products, such as HNE, with respect to glucose. K_M_ values for HNE were found in the low micromolar range (10–30 μM), much lower than those reported for glucose (35–200 mM) [26,27]. In this regard, however, it should be noted that when considering the open form of glucose, the form that is actually transformed by the enzyme, the K_M_ value drops significantly to about 2.5 μM [28].

## 4. Aldose Reductase and Inflammatory Response

One of the main ways of HNE detoxification is the conjugation of HNE with glutathione (GSH), which leads to the formation of the 3-glutathionyl-4-hydroxy-nonanal adduct (GS-HNE) [25]. The formation of GS-HNE may be spontaneous, but the rate of the reaction is greatly enhanced by glutathione S-transferase. GS-HNE formed can be either oxidized or reduced. AKR1B1 can catalyze the reduction of GS-HNE, generating 3-glutathionyl-1,4-dihydroxinonane (GS-DHN). The latter can induce inflammation through the nuclear factor kB (NF-κB) pathway, thus contributing to the onset of secondary diabetic complications. The pro-inflammatory effect of increased concentrations of GS-HNE and GS-DHN could also depend on the activation of macrophages, probably mediated by Toll-like Receptors. In this regard, the levels of TNF-α, a pro-inflammatory cytokine, increase in macrophages after treatment with GS-HNE or GS-DHN [29] (Figure 1).

## 5. Aldose Reductase Inhibitors

Due to the involvement of AKR1B1 in the etiology of diabetic complications, a great effort has been devoted in the last fifty years to the design and the synthesis of molecules able to inhibit AKRB1. More recently, the inhibition of AKR1B1 has been also considered a tool to intervene in the inflammation processes that contribute to the complications in the diabetic patients [30].

The inhibitors of AKR1B1 have multiple structures, but some common features can be identified. ARIs usually present a polar moiety, that interacts with the aforementioned “anion binding pocket” of the enzyme and a hydrophobic moiety, that interacts with the non-polar region of the AKR1B1 active site [6,7,31,32,33].

**Figure 2:**
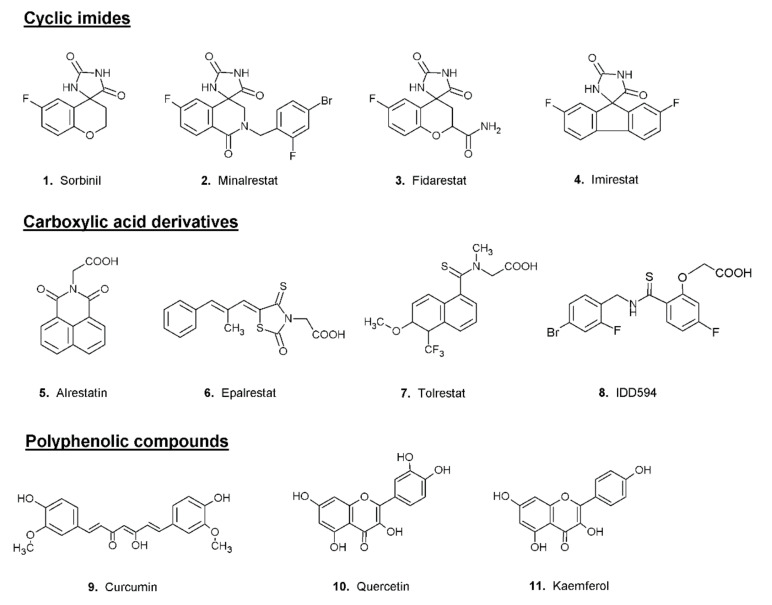
Structures of classical examples of aldose reductase inhibitors.

Most aldose reductase inhibitors can basically be divided into three main classes: compounds that contains cyclic imides, carboxylic-acid derivatives and polyphenolic compounds (Figure 2).

Sorbinil (compound **1** of Figure 2), first introduced by Pfizer, can be considered the progenitor of the class of cyclic imides [34]. Even though this inhibitor is able to efficiently inhibit the in vitro activity of the enzyme, by binding to the active site through the formation of hydrogen bonds [7], the treatment of diabetic patients with Sorbinil showed no significant amelioration of secondary diabetic complications such as polyneuropathy [35]. The ineffectiveness of Sorbinil in diabetic subjects, combined with the occurrence of hypersensitivity reactions to the drug [36], led to the design of new inhibitors, whose structure was based on that of Sorbinil; among them the most effective were found to be Fidarestat, Minalrestat and Imirestat (compounds **2**–**4** of Figure 2) [37].

The structure of Alrestatin (compound **5** of Figure 2), one of the first carboxylic acid derivatives proposed as aldose reductase inhibitors, has been used as a model for the developing of a series of new ARIs. One of the most effective carboxylic derivatives is Epalrestat (compound **6** of Figure 2), a potent AKR1B1 inhibitor capable of lowering the concentrations of fructose and sorbitol produced from the polyol pathway with a consequent reduction of the levels of oxidative stress. Experimental evidence showed the efficacy of Epalrestat in the treatment of diabetic neuropathies and nephropathies [38]. Tolrestat (compound **7** of Figure 2) is another potent AKR1B1 inhibitor of the class of carboxylic acid derivatives. Studies conducted on humans have demonstrated the ability of this inhibitor to improve neuropathy and nephropathy in diabetic subjects [39,40,41]. After its commercialization in several countries, Tolrestat was withdrawn because of serious effects and reduced efficacy in subsequent clinical trials [42]. Among the most studied molecules, tested as AKR1B1 inhibitors, are the compounds of the IDD series. These inhibitors, all designed starting from a common scaffold, are potent and specific with IC_50_ in the low micromolar or nanomolar range [43,44]. The analysis of the crystal structure of the ternary complex of human AKR1B1, NADP^+^ and inhibitor IDD594 (compound **8** of Figure 2) provided valuable information of the molecular mechanism of the opening of the specificity pocket involved in the binding of several AKR1B1 inhibitors. Even though many ARIs of the class of carboxylic acid derivatives show a great affinity for the enzyme in vitro, their potency is highly reduced in vivo. The reduced effectiveness in vivo is related to the very low pK_a_ of these inhibitors, which causes them to be ionized at physiological pH. The negative charge makes it difficult for these compounds to pass through the cell membrane [3].

A great variety of polyphenolic compounds, for example, curcumin, quercetin and kaempferol (compounds **9**–**11** of Figure 2), have been shown to possess a potent inhibitory effect on AKR1B1 activity in various in vitro and in vivo systems [45,46].

The search for new inhibitors of AKR1B1 is an active and constantly evolving field of research and every year several new molecules for the inhibition of AKR1B1 are designed and proposed [11,47,48,49,50].

## 6. Aldose Reductase Differential Inhibitors

Despite the considerable effort devoted in the last decades to the synthesis of AKR1B1 inhibitors, virtually all synthesized compounds failed as drugs for the treatment of diabetic complications. To date, the only drug marketed is Epalrestat, for sale only in eastern countries. The reasons for this failure may be various and range from a reduced bioavailability of the molecules proposed as aldose reductase inhibitors to the onset of important side effects associated with the use of such inhibitors. On the other hand, a possible explanation could lie in the fact that the activity of AKR1B1 under hyperglycemic conditions, in addition to potential harmful effects, can have a beneficial effect, since, as reported above, AKR1B1 is capable of reducing toxic aldehydes, such as HNE and other alkenals and alkanals, which are formed during the lipid peroxidation process. Due to the failure of classical AKR1B inhibitors, a new strategy for inhibiting this enzyme has recently been proposed, based on the possibility of an inhibitory action dependent on the substrate to be transformed [51]. AKR1B1 is a multispecific enzyme since it can catalyze the reduction of hydrophilic as well of hydrophobic substrates (Table 1). However, if we consider the specificity of the enzyme towards its multiple substrates, it appears evident that AKR1B1 is not simply a permissive enzyme but is able to discriminate among different substrates of the same class [52,53]. Emblematic in this regard is the comparison among different isomers and stereoisomers of aldoses (Table 1); in the case of glucose, for instance, the activity of the enzyme towards l-glucose is almost negligible. It is also noteworthy that in the case of glucose and other aldoses the reaction rate of AKR1B1 as a function of substrate concentrations appears biphasic. Believed to be associated with the heterogeneity of the used enzyme preparations supposed to contain two oxidative states of the enzyme [54], the biphasic behavior observed with glucose and other aldose substrates turned out to depend on an incomplete inhibition exerted on the aldehyde reduction by the hemiacetal form of the sugar [26].

**Figure 3:**
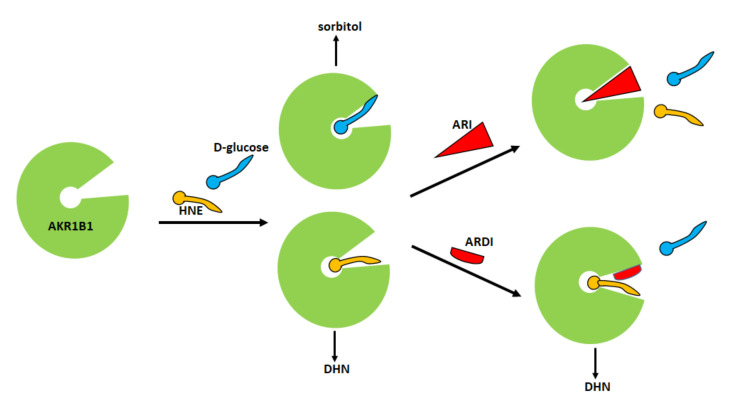
Classical and differential inhibition of AKR1B1. A classic inhibitor of AKR1B1 binds to the enzyme preventing both glucose and HNE from binding. A differential inhibitor binds to the enzyme and prevents glucose from binding but leaves the enzyme’s ability to transform HNE unaffected. HNE: 4-hydroxy-2,3-nonenal; DHN: 1,4-dihydroxy-2,3-nonene.

The very fact that the enzyme can reduce both hydrophobic and hydrophilic aldehydes suggests that different molecules may interact with the enzyme following alternative pathways. Indeed, a molecular modelling study has highlighted the different ways in which the substrates glucose, HNE and GS-HNE interact with the active site of the enzyme [56]. The computational analysis revealed that, in addition to the hydrogen bonds with the catalytic residues H110 and Y48, glucose forms other hydrogen bonds with residues W111 and L300, present in the active site of the enzyme and with the cofactor NADPH. HNE, besides the two hydrogen bonds with the catalytic residues H110 and Y48, seems to induce the opening of the “specificity pocket” that accommodates the hydrophobic tail of HNE. As for GS-HNE, the HNE moiety of the molecule interacts with the “specificity pocket” in a similar way to that of HNE, while the glutathionyl moiety forms hydrogen bonds with V47, W20 and Q49 residues located in the upper part of the active site [56]. The consideration that different molecules may bind to the catalytic site of the enzyme following alternative ways supports the idea that it might be possible to act selectively on the enzyme by blocking its unwanted activities and preserving the advantageous ones. Thus, a useful aldose reductase differential inhibitor (ARDI) should preferentially inhibit the activity of AKR1B1 on glucose and/or GS-HNE, leaving unaltered or less affected the detoxifying action of the enzyme against HNE. Therefore, an ARDI would prevent the diabetic complications related to the activation of the polyol pathway and the pro-inflammatory effect of AKR1B1; on the other hand, it would maintain the detoxification capacity of the enzyme, contributing to the reduction of oxidative stress. The schematic representation of the ARDI action reported in Figure 3 emphasizes the different positioning of the substrate at the active site as the rational base of a possible differential inhibitory action.

As highlighted in a theoretical study, a differential inhibitor of a multispecific enzyme should be able to preferentially bind to the free enzyme, that is, it should behave as a competitive inhibitor, or, alternatively, while being able to bind to both the free enzyme and the enzyme-substrate complex, in a mixed type inhibition, it should still have a significant affinity for the free enzyme [57].

Generally speaking, it is conceivable that, irrespective of the mechanism of action, a very powerful inhibitor, strongly interfering with the key catalytic function of Tyr-48, could hardly act as an ARDI, being unable to discriminate between different substrates.

**Figure 4:**
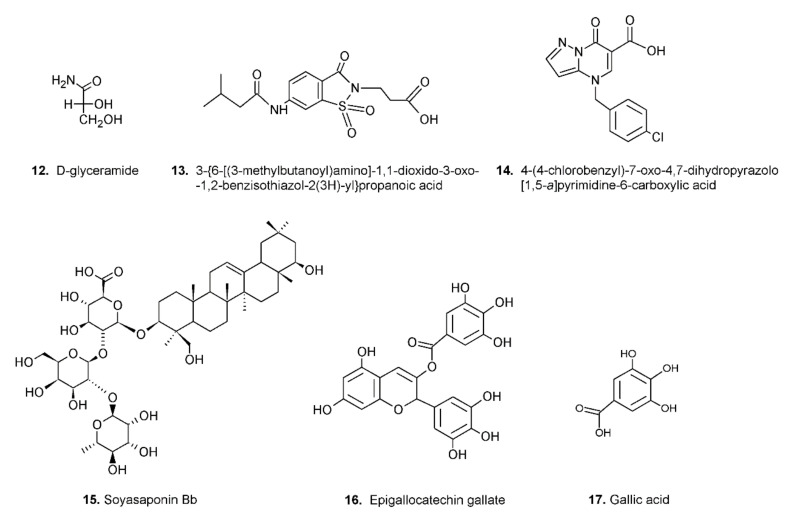
Structures of molecules acting as ARDIs.

Despite the above mentioned restrictions, in a precursor study, clear kinetic evidence was obtained that some molecules are able to preferentially inhibit the reduction of the hydrophilic substrates (e.g., d-glyceramide, compound **12** of Figure 4), while others are more efficient at inhibiting the reduction of the hydrophobic ones (e.g., compound **13** of Figure 4) [51].

Starting from this first experimental evidence, a subsequent screening has been undertaken on a series of compounds previously developed as classical inhibitors of AKR1B1, in order to test the possibility that some of them could act as ARDIs. The possibility that different substrates bind differently to specific parts of the enzyme [56] and the fact that the hemiacetal form of long-chain aldoses can modulate the activity of AKR1B1 [26] has raised the more general question of which substrate should be used in screening studies of AKR1B1 inhibitors. Most inhibition studies use d,l-glyceraldehyde, a triose, which, in the light of the above considerations, cannot be considered the model substrate of the enzyme. Thus, in studies in which molecules for the inhibition of the reduction of d-glucose by AKR1B1 are searched for, d-glucose should be used as the appropriate substrate. However, d-glucose, due to the very high K_M_ of AKR1B1 for this sugar, is a very poor substrate for the enzyme. Therefore, either large amounts of enzyme or extremely high glucose concentrations are required to have acceptable rate measurements. To circumvent the methodological limitations posed by the use of d-glucose, l-idose, the C-5 epimer of d-glucose, which mimics the structure of d-glucose, has been proposed as a good substrate alternative to d-glucose [28] and should be routinely used for searching for ARDIs. Furthermore, the evidence that dimethylsulfoxide (DMSO)—the solvent most frequently used to solubilize compounds during AKR1B1 inhibition studies—affects l-idose and HNE reduction through different inhibition mechanisms [58] highlighted the need to strictly control the solvent concentration especially in studies looking for ARDIs.

The search for ARDIs among some previously described AKR1B1 inhibitors evidenced that classical potent AKR1B1 inhibitors, such as Epalrestat and Sorbinil, showed no ability to differentially inhibit AKR1B1. At the same time, it has been shown that other molecules are able to modulate the activity of the enzyme depending on the specific substrate (l-idose, HNE or GS-HNE adduct). In particular, when screening molecules of a class of acid derivatives of pyrazolo[1,5-*a*] pyrimidine compounds (compound **14** of Figure 4), characterized by the presence of a pyrazolo[1,5-*a*] pyrimidine core and a carboxylic moiety, it has been reported that some compounds in this class can preferentially inhibit the reduction of sugars and GS-HNE adduct with respect to HNE [59]. Explaining the structural features required for a molecule capable of inhibiting a certain mechanism of action while leaving an alternative pathway unaffected is not easy. The extensive structural information available for AKR1B1 is less helpful than one would expect, since it is lacking the most relevant information concerning the reciprocal influence between the substrate and the inhibitor both positioned on the enzyme. At the level of pure speculation, one might hypothesize that compounds able to preferentially inhibit the reduction of HNE over the reduction of glucose are able to somehow block the opening of the specificity pocket that allocates the hydrophobic tail of the HNE. This hypothesis might not be valid in the case of molecules which preferentially inhibit the reduction of glucose. In this case, the picture is further complicated by the presence of the hemiacetal form of glucose that influences the activity of AKR1B1.

Although the possibility of differential inhibition of AKR1B1 has been evidenced using synthetic molecules, the design and synthesis of new drugs for human use is a time-consuming and expensive process. Therefore, alongside the inhibition studies of AKR1B1 conducted using synthetic molecules, other studies have focused on compounds of natural origin. There are numerous examples of plant-derived molecules acting as powerful inhibitors (for a review see [45]). Some of them could also be able to act as ARDIs. Even though the search of ARDIs in extracts from natural sources is not an easy task, since in complex mixtures the presence of AKR1B1 classical inhibitors may have a masking effect on ARDIs, some promising results were obtained. After a first screening of extracts from edible vegetables that suggested the presence of molecules potentially acting as ARDIs [55], some triterpenoid saponins were identified in extracts of seeds of the Zolfino bean landrace as effective AKR1B1 inhibitors; among them, soyasaponin Bb (compound **15** of Figure 4) was shown to be able to differentially inhibit human AKR1B1 [60]. More recently, a study has been carried out on molecules that are present in green tea to verify the possibility that some of them, already reported be AKR1B1 classical inhibitors [61,62], can also act as ARDIs. In this study, it has been shown that epigallocatechin gallate (EGCG), the ester of epigallocatechin and gallic acid, the most abundant catechin in green tea (compound **16** of Figure 4), preferentially inhibits l-idose and GS-HNE reduction with respect to HNE, while gallic acid (GA) (compound **17** of Figure 4), which is still able to inhibit sugar reduction more efficiently than HNE reduction, is less efficient at inhibiting GS-HNE reduction [56]. The molecular modeling study undertaken in order to explain the behavior of EGCG, GA and ECG provided some insight into the molecular interactions that allow some molecules to preferentially inhibit the reduction of l-idose and/or GS-HNE with respect to the reduction of HNE. EGCG interacts with the catalytic site of AKR1B1 in a region predicted to be occupied by the glutathionyl moiety of GS-HNE and could inhibit the reduction of GS-HNE and l-idose mainly by steric hindrance, hampering the binding of GS-HNE to the enzyme and blocking l-idose into the catalytic pocket. As for GA, it can form two strong H-bonds with l-idose, but only one weaker bond with HNE and GS-HNE: therefore, it can be assumed that GA is able to block l-idose more efficiently than HNE and GS-HNE at the level of the catalytic site [56]. To conclude, besides the preliminary information reported above concerning the ability of natural and synthetic molecules to differently affect the reduction of different substrates, and the different positioning of the different substrates suggested by the molecular modelling analysis, at the moment no structural data are available to provide the rationale for the differential inhibition phenomenon or to direct the synthesis and/or the screening towards specific classes of compounds. Unfortunately, even the possible contribution of the presence of hemiacetals present in the case of long-chain aldoses to the phenomenon of differential inhibition is not currently supported by any structural information.

In any case, the search for AKR1B1 differential inhibitors appears to be a new and promising research field. Understanding the structural requirements that allow the differential inhibition of AKR1B1 can first of all contribute to the development of new molecules able to counteract the secondary complications of diabetes without affecting the detoxifying capacity of the enzyme, but it can also contribute to the development of new inhibition strategies for all those multifunctional enzymes capable of recognizing different substrates through different interaction pathways.

## Figures and Tables

**Table 1:** **^a^** List of AKR1B1 substrate examples. The specificity constant K_S_ is reported for each substrate.

Hydrophobic Substrates and Derivatives	*K*_s_ mM^−1^min^−1^	Aldoses	*K*_s_ mM^−1^min^−1^
propanal	5.5	d-glyceraldehyde	1860
butanal	439	l-glyceraldehyde	6840
hexanal	3657	d-threose	114
nonanal	1157	l-threose	276
*trans*-2-pentenal	61.7	d-arabinose	0.24
*trans*-2-nonenal	2058	l-arabinose	10.8
4-hydroxy *trans*-2-pentenal	199	d-xylose	8.4
4-hydroxy *trans*-2-nonenal	921	l-xylose	0.48
*trans*-4-decenal	1108	d-idose	4.2
*cis*-4-decenal	862	l-idose	35.75
4-hydroxy *trans*-2,3-nonenal	2320	d-glucose	0.61
3-glutathionyl-4-hydroxy-nonanal	1376	l-glucose	n.d. **^b^**

**^a^** Adapted from [55]; **^b^** Not determinable; l-glucose is not a substrate up to 30 mM concentration.
